# Dietary nutrient intake study among older adults: baseline Malaysian pure study

**DOI:** 10.1186/s12877-024-05042-w

**Published:** 2024-05-20

**Authors:** Mohd Hasni Ja’afar, Nafiza Mat Nasir, Zaleha Md Isa, Rosnah Ismail, Azmi Mohd Tamil, Noor Hassim Ismail, Farnaza Ariffin, Nurul Hafiza Ab Razak, Najihah Zainol Abidin, Khairul Hazdi Yusof

**Affiliations:** 1 Department of Public Health Medicine, Faculty of Medicine, Universiti Kebangsaan Malaysia, Cheras, Kuala Lumpur, 56000 Malaysia; 2 Department of Primary Care Medicine, Faculty of Medicine, Selangor Branch, Universiti Teknologi MARA (UiTM), Jalan Hospital, Sungai Buloh Campus, Sungai Buloh, Selangor 47000 Malaysia; 3 Department of Diagnostic and Allied Health Science, Faculty of Health and Life Sciences, Management and Science University, Shah Alam, Selangor 40100 Malaysia; 4 Risk Management Unit, Hospital Canselor Tuanku Muhriz, Universiti Kebangsaan Malaysia, Cheras, Kuala Lumpur, 56000 Malaysia

**Keywords:** Older adults, Prevalence, Nutrients, Diet, Nutritional status

## Abstract

**Introduction:**

The older adults (OA) is vulnerable to malnutrition, which may affect their health and quality of life. This study assesses the prevalence of deficiencies in dietary nutrients among the Malaysian OA stratified by residency, genders, socioeconomic status (SES) and body mass index (BMI).

**Methodology:**

A cross-sectional study was conducted, utilizing purposive sampling, recruiting 2,299 Malaysian people aged 60 years old and above who agreed to be interviewed via a comprehensive semi-quantitative food frequency questionnaire. The nutrients intake was calculated based on the Malaysian food composition and US Department of Agriculture food composition databases. Then, the nutrients intake was compared with the Malaysian Recommended Nutrients Intake guidelines, and the prevalence of deficiencies in dietary nutrients were calculated. The median (interquartile ranges) intakes of nutrients were compared between residency (urban and rural), genders (male and female), and SES (low and middle-high) using the Mann-Whitney U test. The differences in nutrient intake between BMI categories (underweight, normal, and overweight) were identified using the Kruskal-Wallis test followed by Dunn’s post hoc test.

**Results:**

The response rate was 70.3% (*n* = 2,299), predominantly were females (50.8%), received primary education (76.6%), were currently married (84.3%), were middle–high SES (57.7%), and had a normal BMI (59.8%). There was a notable inadequate intake prevalence of magnesium (100.0%), manganese (97.9%), zinc (95.6%), vitamin B_6_ (98.4%), potassium (91.0%), calcium (89.3%), vitamin B_12_ (80.2%), vitamin E (91.2%), and vitamin K (81.5%) among Malaysian OA. Additionally, significant differences were observed in nutrients intake levels across gender, residency, SES, and BMI within this population.

**Conclusions:**

This study shows a high prevalence of dietary nutrients deficiency (> 80%) among the Malaysian OA, particularly for magnesium, manganese, potassium, zinc, vitamin B_6_, vitamin E, calcium, vitamin B_12_, and vitamin K. To improve the nutritional status of OA and safeguard against adverse health effects, it is necessary to formulate and execute strategies to enhance their dietary nutrient intakes. The strategies may involve intervention such as nutrient supplementation and promotion of consuming nutrient-rich foods.

## Introduction

The world’s population is aging, and both developed and developing countries are experiencing an increase in the number of older adults (OA). Better healthcare, sanitation, and access to education result in increased longevity, and lower mortality and fertility rates. Thus, according to the World Health Organization (WHO), the proportion of those aged > 60 years old is expected to nearly double from 12 to 22% between 2015 and 2050 [1]. Malaysia is no exception and, by 2030, this country is expected to become an ageing nation with 14% of its population will be aged > 60 years old, and further increase to 24% by the year 2050 [2]. Data from the national census by the Department of Statistics Malaysia (2023) showed an increase in the OA aged ≥ 60 years old from 3.6 million (11.1%) to 3.8 million (11.3%) within less than a year from 2022 till mid of 2023 [3]. As the aging population grows, the burden of diseases among OA rises, posing a challenge to the healthcare systems with high potential of increasing healthcare expenditures of the country in the future [4, 5].

Apart from being physically active and having regular medical check-ups, nutrition or dietary intake plays a crucial role in helping this population either maintain their health or reduce complications due to various health conditions [6–8]. Moreover, OA are more likely to be in an impaired nutritional state and are at higher risk of nutritional deficiency [6, 8]. The nutritional deficiencies among OA were reported worldwide, specifically for calcium, magnesium, vitamin B_6_, vitamin B_12_, and vitamin E in China and the US [9–11]. A previous study done in Malaysia has reported that the OA was nutritionally deficient for, in particular, vitamin E, vitamin B_1_, vitamin B_3_, vitamin B_9_, calcium, and zinc [12]. While another Malaysian study focusing on the OA living in agricultural settlements has found that most of this population does not meet the recommended nutrient intake (RNI) for calcium, vitamin D, and vitamin B_2_ [13]. Several studies have reported that gender, residency area (urban and rural), and socioeconomic status (SES) are associated with nutrition intakes among the OA [9, 10, 12, 13].

To the best of our knowledge, studies on dietary nutrients intake focusing on the Malaysian OA that compare the dietary intakes between residency area (urban and rural), socioeconomic status (SES), and body mass index (BMI) are still scarce. Thus, this study aimed to assess the prevalence of deficiencies in dietary nutrients among the Malaysian OA stratified by residency, gender, SES and BMI to identify the most vulnerable groups that require care and intervention.

## Methodology

### Study design and samples

Data was obtained from the regional sub-study of participants from Malaysia enrolled in the multi-national Prospective Urban Rural Epidemiology (PURE) study, which covers both urban and rural regions. This cohort study baseline data collection was conducted from 2007 to 2008, and the follow-up is ongoing until 2030. The comprehensive study design and samples were described previously [14–18].

Participants were purposely recruited from 90 communities (59 urban and 31 rural areas) in the Peninsular and East Malaysia. With permission from community leaders, health screening and promotion booths were set up in the communities’ assembly halls where interested and eligible participants were briefed about the study. Medical histories were taken, basic physical examinations were conducted and home visits were arranged after obtaining written informed consent from each participant. During the home visits, other individuals living in the same household were invited to join the study. Only the household members intending to continue living in their current home for a further 4 years were selected to ensure the feasibility of long-term follow-up. In this particular paper, we analyzed data from 2,299 participants aged 60 years old and above who provided completed questionnaires during the baseline data collection. Participants with any cardiovascular diseases (e.g., stroke, angina and heart failure) as well as those with cancer were excluded from the data analysis.

All data were obtained through face-to-face interview sessions by a group of well-trained research assistants. Research assistants undergo comprehensive training to ensure standardized data collection methods. Data are electronically transferred to the PHRI for quality control checks.

### Data collection and measurements

Participants’ habitual food intake was recorded using a validated 146-item semi-quantitative food frequency questionnaire (FFQ) [19]. Participants reported the usual portion size (e.g., one egg, one tablespoon, or one cup) of each food item in the FFQ and the average frequency of consumption (in the range of never to more than six times per day). Then, nutrients in terms of total energy, carbohydrate, protein, fat, calcium, copper, iron, magnesium, manganese, phosphorus, potassium, selenium, zinc, vitamin A, vitamin B_6_, vitamin B_12_, vitamin C, vitamin E, and vitamin K intakes were calculated based on the Malaysian and US Department of Agriculture food composition databases, with reference to nutrient databases containing the recipes of mixed dishes [20]. The consumption of nutrient supplements or medicines was not analyzed in this study. The prevalence of deficiencies in dietary nutrients were evaluated using the Recommended Nutrients Intake (RNI) guideline by the Malaysian Ministry of Health (MOH) [21]. The proportion of participants consuming dietary energy and each specific nutrient less than the RNI was evaluated.

A standard set of questionnaires was used to gather information on the individual socio-demographics including age (rounded to the nearest year of birth), gender, residency (urban and rural), socioeconomic status (SES of low and middle–high), education level (primary, secondary, and tertiary) and marital status (currently married and currently unmarried). Type of residency was defined as urban when the areas were occupied by more than 150 residents per square kilometer. Low SES was defined by an income of < RM1000, and those with an income of ≥ RM1000 were defined as middle-high SES. Height was measured using a portable stature meter and the TANITA (BC-558 Ironman®) segmental body composition analyzer was used to measure weight. Body mass index (BMI) was calculated by dividing weight (in kilograms) by height (in meters) squared. BMI for participants aged 60 to 65 years old were categorized as underweight (< 23 kg/m^2^), normal (23 to 28 kg/m^2^), and overweight (> 28 kg/m^2^), while participants aged 66 to 70 years old were categorized as underweight (< 24 kg/m^2^), normal (24 to 29 kg/m^2^), and overweight (> 29 kg/m^2^) [22].

### Statistical analysis

The data were analyzed using SPSS version 26 (IBM, Armonk, NY, USA). The general characteristics of participants were descriptively analyzed and are presented as the median and interquartile range (IQR) for continuous data and frequency (and corresponding percentages) for categorical data. The prevalence of deficiencies in dietary energy and nutrients intake was calculated according to the formula below;

$$\text{deficiency prevalence}=\frac{n}{t}\times 100\%$$

Where;

n = number of participants who reported a dietary intake below the RNI, t = total number of participants.

The Mann–Whitney U test was used to identify the differences in nutrients intake between residency (urban and rural), genders (male and female) and SES (low and middle-high). The Kruskal-Wallis test, followed by Dunn’s post hoc test, was utilized to determine the differences in nutrient intake across BMI categories (underweight, normal and overweight). The statistical significance level was set at *p* < 0.05.

## Results

This study involved 2,299 participants with the proportion of 1,130 (49.2%) males and 1,169 (50.8%) females. The general socio-demographic characteristics of study participants are summarized in Table 1. The majority of the participants received primary education (*n* = 1,759, 76.6%), were currently married (1,933, 84.3%), were middle–high SES (1,258, 57.7%), and were normal BMI (1,374, 59.8%).

**Table 1:** Socio-demographic distribution of participants (*n* = 2,299)

Characteristics	Total	Urban	Rural
	Mean ± SD		
Age (years)	64.3 ± 3.2	64.1 ± 3.1	64.4 ± 3.2
	N(%)		
Gender (*n* = 2,299)			
Male	1130(49.2)	493(43.6)	637(56.4)
Female	1169(50.8)	430(36.8)	739(63.2)
Education level (*n* = 2,295)			
Primary	1759(76.6)	561(31.9)	1198(68.1)
Secondary	471(20.5)	308(65.4)	163(34.6)
Tertiary	65(2.8)	53(81.5)	12(18.5)
Marital status (*n* = 2,293)			
Currently married	1933(84.3)	782(40.5)	1151(59.5)
Currently unmarried	360(15.7)	139(38.6)	221(61.4)
SES (*n* = 2,182)			
Low	924(42.3)	9(1.0)	915(99.0)
Middle-High	1258(57.7)	883(70.2)	375(29.8)
BMI (*n* = 2,299)			
Underweight	469 (20.4)	102(21.7)	367(78.3)
Normal	1374 (59.8)	660(48.0)	714(52.0)
Overweight	456 (19.8)	160(35.1)	296(64.9)

In general, this study found a high prevalence of deficient dietary nutrient intakes among the OA, as shown in Table 2. The intake of macronutrients intake in this population shows that 34.9%, 54.5%, 1.1% and 1.3% of them were inadequate in terms of energy, carbohydrate, protein, and fat, respectively. There was a notable prevalence of deficiencies in the intake of magnesium (100.0%), manganese (97.9%), zinc (95.6%), vitamin B_6_ (98.4%), potassium (91.0%), calcium (89.3%), vitamin B_12_ (80.2%), vitamin E (91.2%), and vitamin K (81.5%).

**Table 2:** Malaysian Recommended Nutrients Intakes (RNI) 2017 for OA and general nutrients intakes with its deficiency prevalence

Nutrients	Recommended Nutrient Intakes (RNI) 2017 for Malaysia		Total dietary nutrients intake	
	Female	Male	Med (IQR)	Below RNI (%)
Energy (kcal/day)	1770	2030	2350.3 (1659.1-3088.8)	34.9
Carbohydrate (g/day)	885^#^	1015^#^	306.5 (230.3–400)	54.5
Protein (g/day)	50	58	97.8 (65.6-136.2)	1.1
Fat (g/day)	49^*^	56^*^	74.9 (47.9-105.5)	1.3
Calcium (mg/day)	1200	1000	601.5 (390.2-840.4)	89.3
Copper (mg/day)	0.9		3.8 (0.2–8.2)	30.4
Iron (mg/day)	8	9	28.0 (18-41.5)	30.7
Magnesium (mg/day)	420^a^, 320^b^	420	32.2 (17-59.8)	100.0
Manganese (mg/day)	1.8	2.3	0.4 (0.2–0.7)	97.9
Phosphorus (mg/day)	700		1467.0 (957-2031.4)	10.7
Potassium (g/day)	4.7		2.5 (1.5–3.5)	91.0
Selenium (µg/day)	23	31^c^, 30^d^	27.0 (14.8–48.5)	48.6
Zinc (mg/day)	4.4^c^, 4.3^d^	6.3^c^, 6.2^d^	1.5 (0.8–2.4)	95.6
Vitamin A (µg/day)	600		1154.9 (644.6-1623.6)	22.5
Vitamin B_6_ (mg/day)	1.5	1.7	0.2 (0.1–0.4)	98.4
Vitamin B_12_ (µg/day)	4.0		1.4 (0.6–2.5)	80.2
Vitamin C (mg/day)	70		111.8 (64.2-171.8)	28.0
Vitamin E (mg/day)	7.5	10	1.6 (0.7-3)	91.2
Vitamin K (µg/day)	55	65	17.1 (4.5–37.8)	81.5

^#^calculated based on 50% of energy intake 1770 (female) and 2030 (male), *calculated based on 25% of energy intake 1770 (female) and 2030 (male), ^a^60-69 years old, ^b^>70 years old, ^c^60-65 years old, ^d^>65 years old

### Assessment of dietary nutrients intake between urban and rural areas

The specific median (IQR) of nutrients intake among the OA in comparison with the RNI stratified by location are presented in Table 3. The intakes of macronutrients in terms of energy, carbohydrate, protein, and fat shows that 39.1%, 57.3%, 0.1%, and 0.3%, respectively, were inadequate among the urban OA. Meanwhile, energy (32.1%), carbohydrate (52.6%), protein (1.8%), and fat (1.7%) intake were inadequate in the rural OA. Deficiencies in nutrient intakes of calcium, manganese, potassium, zinc, vitamin B_6_, vitamin E, and vitamin K were more prevalent among the urban OA (92.0%, 98.6%, 94.6%, 96.6%, 98.9%, 92.3%, and 83.1%, respectively) compared to the rural OA (87.5%, 97.4%, 88.7%, 94.8%, 98.0%, 90.4%, and 80.4%, respectively).

**Table 3:** Dietary nutrients intake and deficiency prevalence among OA in urban and rural (*n* = 2,299)

Nutrients	Urban		Rural	
	Med (IQR)	Below RNI (%)	Med (IQR)	Below RNI (%)
Energy (kcal/day)	2210.4 (1620–2813)	39.1	2475.0 (1708.9-3278.9)	32.1
Carbohydrate (g/day)	286.0 (227.5-360.5)	57.3	326.0 (232.9-429.6)	52.6
Protein (g/day)	93.2 (65.2-125.7)*	0.1	101.1 (66-143.5)*	1.8
Fat (g/day)	71.5 (46.2–98.3)	0.7	76.8 (49.9-112.6)	1.7
Calcium (mg/day)	559.2 (385.1-740.4)*	92.0	646.1 (397-902.3)*	87.5
Copper (mg/day)	3.9 (3.7–8.2)	20.8	3.8 (0.1–8.1)	36.9
Iron (mg/day)	26.5 (19.2–36.9)	20.9	29.2 (16.5–43.3)	37.3
Magnesium (mg/day)	35.3 (20.5–59.5)	100.0	30.2 (13.9–63.6)	100.0
Manganese (mg/day)	0.4 (0.2–0.6)	98.6	0.5 (0.2–0.7)	97.4
Phosphorus (mg/day)	1354.5 (932.6-1843.3)*	8.5	1529.1 (987.1–2152)*	12.3
Potassium (g/day)	2.3 (1.6-3)	94.6	2.6 (1.5–3.7)	88.7
Selenium (µg/day)	27.4 (16.2–43.8)*	48.3	26.8 (14.3–52)*	48.8
Zinc (mg/day)	1.5 (1-2.4)	96.6	1.5 (0.7–2.5)	94.8
Vitamin A (µg/day)	1217.9 (771.1-1551.7)*	17.9	1078.5 (590.2-1692.8)*	25.6
Vitamin B_6_ (mg/day)	0.3 (0.1–0.4)	98.9	0.2 (0.1–0.4)	98.0
Vitamin B_12_ (µg/day)	1.7 (0.7–2.4)*	79.5	1.2 (0.5–2.6)*	80.6
Vitamin C (mg/day)	106.6 (69.6-154.6)*	25.8	114.6 (59.4-183.7)*	29.5
Vitamin E (mg/day)	1.9 (0.9-3)	92.3	1.4 (0.6–3.1)	90.4
Vitamin K (µg/day)	22.1 (9.2–35.9)*	83.1	13.1 (3-44)*	80.4

*Significant at *p*-value < 0.05

### Assessment of the dietary intake of energy and nutrients between genders

Table 4 summarizes the specific median (IQR) of nutrients intake among the OA in comparison with the RNI stratified by gender. The dietary energy and carbohydrate intakes showed a high prevalence of deficiency among the male OA (37.3% and 56.5%, respectively) compared to the female OA (32.6% and 52.5%, respectively). Dietary protein and fat intakes showed a higher prevalence of deficiency among the female OA (1.5% and 1.6%, respectively) compared to the male OA (0.8% and 1.0%, respectively). Additionally, the prevalence of calcium, magnesium, vitamin B_6,_ and vitamin B_12_ deficiencies was higher among the female OA (92.1%, 98.3%, 98.8%, and 80.9%, respectively) compared to the male OA (86.4%, 97.4%, 98.0%, and 79.4%, respectively).

**Table 4:** Dietary nutrients intake and deficiency prevalence among OA in different genders (*n* = 2,299)

Nutrients	Female		Male	
	Med (IQR)	Below RNI (%)	Med (IQR)	Below RNI (%)
Energy (kcal/day)	2337.6 (1607.1-3083.9)	32.6	2392.7 (1732.3-3094.4)	37.3
Carbohydrate (g/day)	304.8 (221.2-402.4)*	52.5	307.5 (238.3–398)*	56.5
Protein (g/day)	96.8 (64-135.3)	1.5	99.1 (66.8-137.3)	0.8
Fat (g/day)	71.7 (46.2-103.3)	1.6	77.8 (49.5-108.3)	1.0
Calcium (mg/day)	606.8 (389.4-856.6)*	92.1	597.8 (392.2-828.2)*	86.4
Copper (mg/day)	3.8 (0.2–8.1)*	30.6	3.8 (0.2–8.2)*	30.3
Iron (mg/day)	27.8 (17.6–41.7)*	28.7	28.2 (18.2–41)*	32.7
Magnesium (mg/day)	32.0 (16.1–59.6)	98.3	32.9 (17.7–63)	97.4
Manganese (mg/day)	0.4 (0.2–0.7)	100.0	0.4 (0.2–0.7)	100.0
Phosphorus (mg/day)	1438.3 (918.9-2031.4)*	12.0	1477.4 (995.8-2035.5)*	9.5
Potassium (g/day)	2.4 (1.5–3.5)	90.9	2.5 (1.5–3.5)	91.2
Selenium (µg/day)	26.5 (14.1–47)*	44.4	28.0 (15.4–49.7)*	52.9
Zinc (mg/day)	1.4 (0.8–2.4)*	93.6	1.5 (0.8–2.5)*	97.6
Vitamin A (µg/day)	1143.6 (616.1-1618.2)	23.7	1167.4 (668.1-1629.6)	21.2
Vitamin B_6_ (mg/day)	0.2 (0.1–0.4)	98.8	0.2 (0.1–0.4)	98.0
Vitamin B_12_ (µg/day)	1.3 (0.5–2.4)	80.9	1.4 (0.6–2.7)	79.4
Vitamin C (mg/day)	113.4 (67.4-172.7)*	27.0	110 (61.8-170.7)*	29.0
Vitamin E (mg/day)	1.5 (0.7–2.9)	89.0	1.6 (0.7–3.1)	93.5
Vitamin K (µg/day)	16.9 (4.5–38)*	79.8	17.4 (4.5–37)*	83.2

*Significant at *p*-value < 0.05

### Assessment of dietary intakes of energy and nutrients between different SES

The specific median (IQR) intakes of nutrients among the OA in comparison with the RNI stratified by SES are shown in Table 5. Surprisingly, the deficient intakes of energy and carbohydrate were more prevalent among middle–high SES (38.5% and 55.2%, respectively) compared to low SES (31.2% and 52.7%, respectively). Meanwhile, the deficient intake of protein and fat were more prevalent among low SES (1.6% and 2.5%, respectively) than middle–high SES (0.8% and 0.6%, respectively). Interestingly, deficient intakes of calcium, potassium, zinc, vitamin B_6_, vitamin B_12_, vitamin E, and vitamin K were more prevalent among the middle–high SES (90.4%, 94.3%, 96.7%, 98.6%, 81.6%, 92.3%, and 84.5%, respectively) compared to the low SES (87.9%, 87.2%, 94.7%, 97.8%, 79.0%, 90.3%, and 77.8%, respectively).

**Table 5:** Dietary nutrients intake and deficiency prevalence among OA in different SES (*n* = 2,182)

Nutrients	Low		Middle-High	
	Med (IQR)	Below RNI (%)	Med (IQR)	Below RNI (%)
Energy (kcal/day)	2544.3 (1716.7-3301.7)*	31.2	2197.2 (1625.5-2901.1)*	38.5
Carbohydrate (g/day)	339 (239.9-431.7)	52.7	288.1 (223.5-374.5)	55.2
Protein (g/day)	103.8 (67.7-144.9)	1.6	92.6 (64.4-128.1)	0.8
Fat (g/day)	79.3 (50.1-112.6)*	2.5	71.4 (46.3-100.8)*	0.6
Calcium (mg/day)	662.2 (410.2-892.6)	87.9	565.1 (377.8-769.8)	90.4
Copper (mg/day)	3.8 (0.1–8.1)	34.5	3.8 (0.3–8.2)*	27.8
Iron (mg/day)	32.2 (18-44.8)*	39.5	25.5 (17.8–37.1)*	24.0
Magnesium (mg/day)	34.5 (16.4–66.4)	100.0	30.9 (17.3–57.1)	100.0
Manganese (mg/day)	0.4 (0.2–0.7)	97.8	0.4 (0.2–0.7)	97.7
Phosphorus (mg/day)	1556.5 (1010.7-2195.7)	11.9	1351.8 (922.6-1890.7)	10.0
Potassium (g/day)	2.8 (1.6–3.8)*	87.2	2.2 (1.5–3.1)*	94.3
Selenium (µg/day)	29.3 (15.7–52.1)*	45.5	25.3 (13.7–44)*	51.5
Zinc (mg/day)	1.5 (0.8–2.5)	94.7	1.4 (0.8–2.4)	96.7
Vitamin A (µg/day)	1211.7 (614.7-1925.9)*	24.4	1133.4 (661.9-1516.7)*	21.5
Vitamin B_6_ (mg/day)	0.2 (0.1–0.5)	97.8	0.2 (0.1–0.4)	98.6
Vitamin B_12_ (µg/day)	1.4 (0.6–2.8)*	79.0	1.3 (0.5–2.4)*	81.6
Vitamin C (mg/day)	119.4 (64-184.6)*	27.6	104.6 (62.9–158)*	29.1
Vitamin E (mg/day)	1.7 (0.7–3.4)	90.3	1.5 (0.7–2.9)	92.3
Vitamin K (µg/day)	17.6 (3.9–53.9)*	77.8	16.5 (4.8–35.9)*	84.5

*Significant at *p*-value < 0.05

### Assessment of dietary intakes of energy and nutrients between different BMI

Table 6 summarizes the specific median (IQR) of nutrients intake among the OA in comparison with the RNI stratified by BMI. The dietary energy, protein and fat intakes showed a higher prevalence of deficiency among the underweight OA (38.6%, 1.9% and 3.2%, respectively) compared to normal (34.3%, 0.9%, and 0.7%, respectively) and overweight (33.3%, 0.9%, and 1.1%, respectively). Meanwhile, the deficient intake of carbohydrate was more prevalent among normal (57.7%) compared to underweight (49.9%) and overweight (49.8%). The overweight OA had the highest prevalence of calcium deficiency (90.4%) compared to underweight (87.4%) and normal (89.5%) OA. Deficient intakes of copper, iron, phosphorus, potassium, selenium, sodium, zinc, vitamin A, vitamin B_6_, vitamin B_12_, vitamin C, vitamin E, and vitamin K were more prevalent among the underweight OA compared to normal and overweight OA.

**Table 6:** Dietary micronutrients intake and the prevalence of deficiency among OA in different BMI (*n* = 2299)

Nutrients	Underweight		Normal		Overweight	
	Med (IQR)	Below RNI (%)	Med (IQR)	Below RNI (%)	Med (IQR)	Below RNI (%)
Energy (kcal/day)	2299.8 (1574.1-3102.8)	38.6%	2339.4 (1672.4-3078.5)	34.3%	2417.0 (1666.5-3113.9)	33.3%
Carbohydrate (g/day)	306.4 (219.3-402.4)	49.9%	301.3 (231.0-392.1)	57.7%	317.7 (232.0-406.6)	49.8%
Protein (g/day)	89.2 (58.5-132.3)^α^	1.9%	99.8 (67.5-137.8)^α^	0.9%	99.9 (66.2-135.7)	0.9%
Fat (g/day)	71.3 (44.1-106.1)	3.2%	76.1 (49.9-105.2)	0.7%	73.4 (47.4-105.7)	1.1%
Calcium (mg/day)	548.7 (343.0-829.4)	87.4%	604.7 (396.5-841.5)	89.5%	621.6 (416.2-846.7)	90.4%
Copper (mg/day)	2.0 (0.1–8.1)^α,β^	41.8%	3.8 (0.5–8.2)^α^	25.9%	3.8 (0.1–8.2)^β^	32.2%
Iron (mg/day)	24.4 (14.3–39.5)^α,β^	37.7%	28.8 (19.0-41.6)^α^	23.5%	29.0 (17.9–42.8)^β^	23.7%
Magnesium (mg/day)	23.3 (10.7–46.3)^α,β,γ^	100%	37.2 (20.1–70.6)^α,γ^	100%	29.9 (16.0-56.9)^β,γ^	100%
Manganese (mg/day)	0.4 (0.1–0.6)	97.9%	0.4 (0.2–0.7)	97.9%	0.4 (0.2–0.6)	97.8%
Phosphorus (mg/day)	1356.5 (848.6–2010.0)	16.2%	1485.6 (994.0-2037.3)	8.7%	1473.0 (983.6-2016.2)	10.5%
Potassium (g/day)	2.1 (1.3–3.3)^α,β^	91.0%	2.6 (1.6–3.5)^α^	90.0%	2.6 (1.6–3.5)^β^	89.7%
Selenium (µg/day)	23.2(10.8–40.2)^α^	55.2%	29.5 (16.8–53.6)^α,γ^	49.3%	25.6 (14.2–44.3)^γ^	47.4%
Sodium (mg/day)	3945.8 (2375.3–6389.0)^α,β,γ^	10.7%	5024.9 (2987.6-9307.5)^α,γ^	7.0%	4361.8 (2622.5-7302.8)^β,γ^	8.3%
Zinc (mg/day)	1.2 (0.6-2.0)^α,β,γ^	96.2%	1.6 (1.0-2.6)^α,γ^	95.5%	1.4 (0.8–2.4)^β,γ^	96.1%
Vitamin A (µg/day)	949.4 (504.5-1555.2)^α^	32.2%	1221.1 (725.4-1653.9)^α,γ^	18.6%	1069.7 (617.2-1605.2)^γ^	24.1%
Vitamin B_6_ (mg/day)	0.2 (0.1–0.3)^α,β,γ^	98.9%	0.3 (0.1–0.5)^α,γ^	98.1%	0.2 (0.1–0.4)^β,γ^	98.7%
Vitamin B_12_ (µg/day)	1.0 (0.4-2.0)^α,β,γ^	89.8%	1.7 (0.7–3.4)^α,γ^	76.1%	1.2 (0.5–2.4)^β,γ^	82.5%
Vitamin C (mg/day)	87.2 (49.2-163.5)^α,β^	36.9%	117.9(68.4-171.7)^α^	26.1%	120.1 (70.5-180.5)^β^	24.8%
Vitamin E (mg/day)	1.1 (0.4–2.2)^α,β,γ^	95.3%	1.9 (0.9–4.3)^α,γ^	89.3%	1.4 (0.6–2.9)^β,γ^	92.5%
Vitamin K (µg/day)	6.0 (1.9–23.5)^α,β,γ^	91.3%	23.1 (7.5–54.4)^α,γ^	77.7%	16.1 (3.8–35.9)^β,γ^	82.9%

^α,β,γ^Dunn’s test with significant *p*-value < 0.05, ^α^Significant Underweight-Normal median differences, ^β^Significant Underweight-Overweight median differences, ^γ^Significant Overweight-Normal median differences

## Discussion

This study assessed the prevalence of deficiencies in dietary nutrients among the Malaysian OA stratified by residency, gender, SES, and BMI. The general findings showed that there was an outstanding deficiency of magnesium (100.0%), manganese (97.9%), potassium (91.0%), zinc (95.6%), vitamin B_6_ (98.4%), vitamin E (91.2%), calcium (89.3%), vitamin B_12_ (80.2%), and vitamin K (81.5%) among Malaysian OA. The median (IQR) intake of magnesium was 32.2 (17.0–59.8) mg/day, which is extremely low compared to the RNI (420 mg/day for males aged > 60 years old and females aged 60–69 years old, 320 mg/day for females aged > 70 years old). Dietary manganese intake in this population was 0.4 (0.2–0.7) mg/day, which is also extremely low compared to the RNI (1.8 mg/day for females and 2.3 mg/day for males). The dietary intake of vitamin B_6_ among this population was 0.2 (0.1–0.4) mg/day, which is lower than the 1.5 mg/day (females) and 1.7 mg/day (males) set by the RNI. Also, dietary vitamin E intake was 1.6 (0.7–3.0) mg/day, which is lower than the RNI of 7.5 mg/day for females and 10.0 mg/day for males. Dietary potassium intake was 2.5 (1.5–3.5) g/day among the OA, that is below the RNI (4.7 g/day) by half. The findings were coherent with Liu et al. who reported deficiencies of vitamin B_6_ (95.1%) and vitamin B_12_ (81.8%) among the OA in China [9]. Meanwhile, the Chinese OA were reported to have a magnesium deficiency of 67.5%, which was lower than reported in this study (100.0% deficiency) [9]. In terms of calcium deficiency, Zamzuri et al. have reported that the Malaysian OA, specifically in Kuantan, has a higher prevalence of deficiency (92.4%) than this study [23]. Similarly, the Chinese OA also had a higher prevalence of calcium deficiency (98.2%) compared to this study (89.3%) [9].

There is compelling evidence linking nutrient deficiencies, such as magnesium, manganese, potassium, zinc, vitamin B_6_, vitamin E, calcium, vitamin B_12_, and vitamin K, to various diseases like hypertension, type 2 diabetes mellitus, cardiovascular diseases, asthma, depression, impaired immunity, cognitive failure (Alzheimer’s disease and other dementia syndromes), psychiatric disorders, muscular diseases, bone fragility, and cancer [24–32]. Previous research has attributed the failure of OA to meet their nutrients needs to factors such as dietary habits, medical conditions (e.g., impaired intestinal absorption due to ageing or medicinal drugs consumption), social status (community dweller or care center residents), psychological factors (e.g., reduced appetite), geographical location, and socioeconomic status [9, 10, 24, 26, 30, 33, 34]. Furthermore, Shahrin et al. concluded that convenience of food preparation has been identified as a factor influencing food choices among the OA [35]. Hence, these may explain on the dietary pattern observed among the Malaysian OA, characterized by high energy intake with moderate carbohydrates, high protein, and fat, but failed to fulfill most of the nutrient requirements. These findings suggest that Malaysian OA tend to choose energy-dense foods over nutrient-dense ones. A nutrient profiling study conducted in Malaysia indicates that energy-dense foods contain less nutrients, and vice versa [36].

This study shows a significantly lower intake of protein, calcium, phosphorus and vitamin C among the urban compared to the rural OA. In contrast, there was a significantly lower intake of selenium, vitamin A, vitamin B_12_, and vitamin K among the rural compared to the urban OA. The urban OA had a significantly lower intake of calcium compared to the rural OA with notable deficiencies among both the urban and rural OA (92.0% and 87.5%, respectively). This finding contradicts those among the OA in China that showed a significantly higher intake of calcium among urban than rural, with also a notable high calcium deficiency in urban and rural areas (96.5% and 99.3%, respectively) [9]. Meanwhile, this study shows that vitamin B_12_ intake among the rural OA is significantly lower than among the urban OA and the deficiency was more prevalent among the rural OA (80.6%) compared to urban OA (79.5%). This finding is in agreement with Liu et al. who reported that vitamin B_12_ intake by the rural OA was significantly lower than the urban OA intake [9]. Similarly, the study also found that vitamin B_12_ deficiency among the rural OA (84.8%) was more prevalent than their urban counterpart (73.1%) [9].

This study shows a significant difference between genders in dietary nutrient intakes regarding carbohydrates, calcium, iron, phosphorus, selenium, zinc, vitamin C, and vitamin K. The male OA had a significantly higher carbohydrate intake than the female OA but the percentage of carbohydrate deficiency in males was higher (56.5%) than in females (52.5%). The patterns are similar to other studies conducted among the OA in Malaysia and China [9, 12, 13]. This study also found that the female consumed a significantly higher amount of calcium compared to the male OA and calcium deficiency was more prevalent among females (92.1%) than males (86.4%). The calcium deficiency was in line with the studies by Liu et al. and Fakhruddin et al. who reported that the prevalence of calcium deficiency in both genders was more than 90.0% [9, 12]. In contrast, the Japanese OA who lived alone in communities recorded only 37.7% deficiency among females and 46.7% deficiency among males [34].

This study reveals that nutrient intake among the OA of middle–high SES elderly were significantly lower compared to the OA of low SES in terms of energy, fat, iron, potassium, selenium, vitamin A, vitamin B_12_, and vitamin K. The energy intake of the middle–high SES was significantly lower compared to the low SES OA. Energy deficiency was more prevalent among the middle–high SES (38.5%) than low SES (31.2%) OA. This trend is similar to the OA in China [10]. Fat intake in this study also showed that the middle–high SES had a significantly lower intake compared to the low SES OA, but fat deficiency was more prevalent among the low SES (2.5%) than the middle–high SES OA (0.6%). A study done by Zhu et al. also reported that the high SES OA in China consumed a significantly lower amount of fat compared to the low SES OA [10].

This study found no significant differences in energy and macronutrient intake across the three BMI groups, except for protein. Protein intake among the underweight OA was significantly lower compared to normal OA. The underweight OA also had significantly lower nutrient intake of magnesium, potassium, zinc, vitamin B_6_, vitamin B_12_, vitamin E, and vitamin K, with a high prevalence of inadequacy (> 80%) compared to their counterparts. A similar trend was observed among overweight OA, showing significantly lower nutrient intake of magnesium, zinc, vitamin B_6_, vitamin B_12_, vitamin E, and vitamin K, with high prevalence of inadequacy (> 80%) compared to the OA with a normal BMI. A study conducted among OA in the US from 2011 to 2014 also reported significant mean differences in intake between normal, overweight and obese OA, with a higher prevalence of inadequate intake of magnesium, vitamin B_6_, vitamin C, and vitamin E among overweight and obese OA compared to those with normal BMI [11]. A study done in Malaysia found that 69.1% of OA with unsatisfactory BMI (underweight, overweight and obese) had a poor perception of oral health compared to those with a normal BMI [37]. Furthermore, OA with unsatisfactory BMI also reported more frequent food restriction, biting, and chewing problems than those with a normal BMI [37]. This may explain the differences in nutrient intakes observed across the BMI categories, suggesting that OA with normal BMI had better nutrient intakes compared to those who were underweight or overweight.

This study highlights the seriousness of magnesium deficiency among the Malaysian OA since the prevalence was 100.0%. The main sources of dietary magnesium are green leafy vegetables, legumes, and whole grains, which this study population always failed to meet the minimum required intake [14, 38, 39]. This study also shows that calcium deficiency was more prevalent among the urban and female OA compared to their other counterparts. The main sources of calcium are milk and dairy products, which are not widely consumed by Malaysians due to high lactose intolerance (> 80%) among the adult population [21, 38, 40]. Additionally, Zamzuri et al. have revealed that the main source of dietary calcium intake among the Malaysian OA was from non-dairy sources [23]. Furthermore, a study done by Kasim et al. showed that powdered milk was ranked sixth among top ten food items consumed daily by Malaysian females in 2003, but it was not listed in 2014 [41]. Similarly, powdered milk was listed as number seven in the top ten food items consumed daily by urban Malaysian adults in 2003, but was unlisted in 2014 [41].

Furthermore, the prevalence of vitamin B_12_ deficiency was higher among the rural and middle–high SES OA than their counterparts. The source of vitamin B_12_ is mainly from animal products, such as meat, fish, eggs, dairy products, and shellfish [21, 42]. The findings of this study are in line with a previous study in Malaysia in 2003 to 2014 that found urban adults consumed a greater variety of animal products compared to the rural adults [41]. Ironically, middle–high SES OA were more prone to vitamin B_12_ deficiency as well as potassium and vitamin K deficiency, compared to the low SES OA. Potassium is abundant in both plant and animal products, such as legumes, nuts, potatoes, bananas, and fishes [21, 43] while vitamin K is mainly from green leafy vegetables [21, 44]. Thus, these findings indicate that the ability to choose healthy foods supersedes the affordability issue among the Malaysian OA. These results contradict those of Zhu et al. who concluded that the low SES OA in China have a significantly lower vitamin status compared to the high SES OA, and that the relationship between vitamin status and SES is mediated by diet quality [10]. In our study population, the majority of the middle–high SES OA resided in urban areas (Table 1). Typically, urban residents are more likely to consume modern food which is a highly processed food and convenient to be prepared, or to eat out, which contains more animal products and less of vegetables and fruits; rural residents, in contrast, depend on traditional diets that are often home-cooked using self-sustained livestock, vegetables, and fruits [38, 40]. Although urbanization has shifted the traditional diet to a modern diet regardless of area [38, 40], the rural OA may still have access to home-grown vegetables and fruits for free or at a cheaper price compared to the urban OA.

There were several limitations noted in this study. First, we used FFQ, which were prone to over or underestimating nutrient intake due to recall bias. However, we only included data with a plausible energy intake in the range of 500-5,000 kcal to overcome this limitation. Although a previous study stated that individuals consuming an adequate diet according to the RNI will have a lower the risk of nutrient deficiency compared to those with an inadequate diet [9], future studies were suggested to analyze biomarkers of nutrients in serum for a more accurate description of the nutritional status of the study population. Second, due to the nature of the cross-sectional study design, any causal inference between location, SES, BMI and deficiencies in dietary nutrients could not be elucidated. However, this study provides insight into preventable deficiencies in dietary nutrients among the Malaysian OA regarding a wide variety of macronutrients, micronutrients, and trace elements, which are often not reported by studies conducted in Malaysia.

## Conclusion

This study indicates that the prevalence of deficiencies in dietary nutrients among the Malaysian OA was high, with a prevalence of > 80%, especially for magnesium, manganese, potassium, zinc, vitamin B_6_, vitamin E, calcium, vitamin B_12_, and vitamin K. In term of residency, the urban OA were more susceptible to calcium and vitamin K deficiency, while the rural OA were prone to have vitamin B_12_ deficiency. In addition, the middle–high SES OA were more likely to have potassium, vitamin B_12_, and vitamin K deficiencies compared to the low SES OA. A higher prevalence of inadequate nutrient intakes (copper, iron, phosphorus, potassium, selenium, sodium, zinc, vitamin A, vitamin B_6_, vitamin B_12_, vitamin C, vitamin E and vitamin K) was observed among underweight OA compared to those with normal and overweight BMI. Besides, the male OA were more vulnerable to zinc and vitamin K deficiency, whereas the female OA were susceptible to calcium deficiency. Therefore, it is a significant challenge for healthcare professionals and policymakers to address this issue. Interventions like nutrient supplementation and promoting nutrient-dense food need to be carefully designed in a manner that can be easily accepted and adapted by the OA and their subpopulations. However, changing lifestyles and habits in the later stages of life can be a bit tricky. We suggest that interventions promoting healthy eating should be initiated at an earlier age to maintain health and quality of life during the golden years.

## Data Availability

The data that support the findings of this study are available from PHRI, but restrictions apply to the availability of these data, which were used under license for the current study, and so are not publicly available. Data is however available from the corresponding author (Nafiza Mat Nasir @drnafiza@uitm.edu.my) upon reasonable request and with permission of PHRI.
